# Size Dependence of Gold Nanorods for Efficient and Rapid Photothermal Therapy

**DOI:** 10.3390/ijms25042018

**Published:** 2024-02-07

**Authors:** Wei Zhou, Yanhua Yao, Hailing Qin, Xiaobo Xing, Zongbao Li, Min Ouyang, Haihua Fan

**Affiliations:** 1 Guangdong Provincial Key Laboratory of Nanophotonic Functional Materials and Devices, School of Information and Optoelectronic Science and Engineering, Guangdong Basic Research Center of Excellence for Structure and Fundamental Interactions of Matter, South China Normal University, Guangzhou 510006, China18617170689@163.com (Y.Y.);; 2 Technology & Centre for Optical and Electromagnetic Research, South China Academy of Advanced Optoelectronics and National Center for International Research on Green Optoelectronics, South China Normal University, Guangzhou 510006, China; xingxiaobo@scnu.edu.cn; 3 Ministry of Education Key Laboratory of Textile Fiber Products, School of Materials Science and Engineering, Wuhan Textile University, Wuhan 430200, China; zongbaoli1982@163.com

**Keywords:** size-dependent effects, gold nanorod, surface plasmon resonance, photothermal, cancer

## Abstract

In recent years, gold nanomaterials have become a hot topic in photothermal tumor therapy due to their unique surface plasmon resonance characteristics. The effectiveness of photothermal therapy is highly dependent on the shape and size of gold nanoparticles. In this work, we investigate the photothermal therapeutic effects of four different sizes of gold nanorods (GNRs). The results show that the uptake of short GNRs with aspect ratios 3.3–3.5 by cells is higher than that of GNRs with aspect ratios 4–5.5. Using a laser with single pulse energy as low as 28 pJ laser for 20 s can induce the death of liver cancer cells co-cultured with short GNRs. Long GNRs required twice the energy to achieve the same therapeutic effect. The dual-temperature model is used to simulate the photothermal response of intracellular clusters irradiated by a laser. It is found that small GNRs are easier to compact because of their morphological characteristics, and the electromagnetic coupling between GNRs is better, which increases the internal field enhancement, resulting in higher local temperature. Compared with a single GNR, GNR clusters are less dependent on polarization and wavelength, which is more conducive to the flexible selection of excitation laser sources.

## 1. Introduction

Gold nanorods (GNRs) have wide biomedical applications because of their unique surface chemistry and electronic and optical properties. The surface plasmon resonance characteristics of GNRs can effectively achieve energy localization [1,2,3,4]. The low specific heat capacity of gold makes it easy for gold nanorods to heat up and facilitate heat transfer. These advantages make GNRs a popular material for cancer photothermal therapy. Upon surface plasmon formation, nonradiative relaxation occurs through electron–phonon and phonon–phonon coupling, efficiently generating localized heat that can be transferred to the surrounding environment [5,6,7,8]. The photothermal conversion efficiency can be effectively adjusted by changing the morphology of colloidal gold nanoparticles [9,10]. The use of gold nanorods for plasmonic photothermal therapy has other advantages, including that the absorption peak can be located in the transparent window band of the human body [11,12], high photothermal conversion efficiency [13,14], low biotoxicity [15] and easy surface modification [16,17,18].

At present, the research on GNR photothermal therapy mainly focuses on the following aspects: 1. tailored with targeting moieties on the surface of gold nanoparticles in order to target cancer cells; 2. combine photothermal therapy with other therapeutic strategies to achieve synergistic therapy; 3. change the shape of gold nanoparticles to achieve higher photothermal conversion efficiency. At present, the commonly used gold nanoparticles are gold nanospheres [19,20], gold nanorods [21,22], gold nanostars [23,24], gold nanocages [25,26], gold nanoshells [27,28], gold nanobipyramids [29,30], etc. Although there are a relatively large number of reports comparing the photothermal therapeutic effect of gold nanoparticles with different shapes, there is a lack of reports on the photothermal therapeutic effect of gold nanoparticles with the same shapes but different sizes. In fact, the size of gold nanoparticles can influence the photothermal conversion efficiency in a great extent. Jiang [31] studied the photothermal characteristics of gold nanospheres with diameters ranging from 5 to 50 nm; the photothermal conversion efficiency increased from 0.65 to 0.8 as the diameters decreased from 50 nm to 5 nm. Chen [32] studied the biological toxicity of gold nanospheres with different sizes to mice. Mackey [33] proposed that if the size of the gold nanorod is too large, the proportion of absorption in the extinction will decrease; when the size of the gold nanorod is too small, although the proportion of absorption in the extinction will increase, the extinction will decrease, and the absorption will also decrease. Therefore, studying the size effect of gold nanorods is helpful to optimize the photothermal treatment effect. In previous works, temperature increases are often approximated to electric field enhancements in simulation, and many studies only simulate one or two GNRs; there are few simulations for clusters consisting of many GNRs in cell vesicles, which limits a deeper understanding of the therapeutic effects of different sizes of gold rods.

In addition, the type of laser source is also a point worth considering, usually using a milliwatt continuous laser for photothermal treatment. However, pulsed laser photothermal treatment can further reduce the laser power required to induce cell death. In our previous reported work [34], we confirmed that the continuous laser energy required for photothermal treatment of HepG2 cells with GNRs is 6.7 mW, and the energy required for a femtosecond laser in the same situation is reduced to 1.7 mW. Wu [35] proved that QGY cells loaded with GNRs have no obvious killing effect when 20 W/mm^2^ continuous light is used to irradiate them, while a femtosecond pulsed laser with the same power could kill 90% of QGY cells. According to the TEM images, when the femtosecond laser is irradiated, the rate of energy transfer to the GNRs is much higher than the rate of energy loss in heat dissipation, so the temperature of GNRs will be heated to above the melting point instantly, triggering the explosion of the GNRs. However, when continuous light is used, although the temperature will rise, the rate of energy transfer to the gold rod will be very small, resulting in the GNRs not exploding. Tong [36] studied the effect of GNRs adsorbed on the surface of the cell membrane, and in the cytoplasm during photothermal therapy, he found that the power threshold of cell damage caused by a femtosecond laser is ten-times lower than that of a continuous laser, whether GNRs are on the surface of the cell membrane or in the cytoplasm.

In this work, we present both the theoretical and experimental results of photothermal therapy with GNRs at four aspect ratios, GNR-1 (3.3), GNR-2 (3.5), GNR-3 (4.1), GNR-4 (5.4). The results show that the number of GNRs taken by HepG2 cells, with aspect ratios of 3.3, 3.5, 4.1 and 5.4 are 4978, 3432, 2358 and 768, increase with the decrease in aspect ratio. After the incident femtosecond pulse laser energy is further changed in a range of 28–138 pJ, the cell death time is studied after 20 s of irradiation. All the experimental results show that the cell death time is shortened with increasing laser energy. For GNRs with aspect ratios of 3.3, 3.5, 4.1 and 5.4, the lowest single pulse cycle energy required to kill cells is 28 pJ, 35 pJ, 69 pJ and 52 pJ. The shorter the aspect ratio, the less energy required to kill cells. Through transmission electron microscopy, it is found that GNRs form clusters in vesicles after being taken up by cells, which is in line with a previous work [37]. We use a dual-temperature model to study the interaction between the laser and clusters. Compared with a single GNR, the absorption spectrum of GNR clusters becomes wider and redshifted, and the absorption cross-section of the clusters increases. By comparing the photothermal simulation results of GNR-1 to GNR-4, although single large-size GNRs had a larger absorption cross-section, the arrangement of large-size GNR-3 and GNR-4 is less compact than that of GNR-1 and GNR-2 after forming clusters. The temperature increasement of GNR-1 clusters and GNR-2 clusters is higher than that of GNR-3 clusters and GNR-4 clusters. Careful analysis shows that because the plasma coupling between adjacent GNRs in the GNR-1 and GNR-2 clusters changes the internal field enhancement of each GNR in the clusters, the probability of obtaining a great internal field enhancement is greatly increased, and the temperature associated with the internal field enhancement is also increased. Therefore, the high photothermal conversion ability of a single GNR is not equal to better photothermal treatment after forming clusters. Our study showed that the photothermal treatment effect of small-sized GNRs is better than that of large-sized GNR after forming clusters.

## 2. Results

### 2.1. Characterization of GNRs

**Figure 1:**
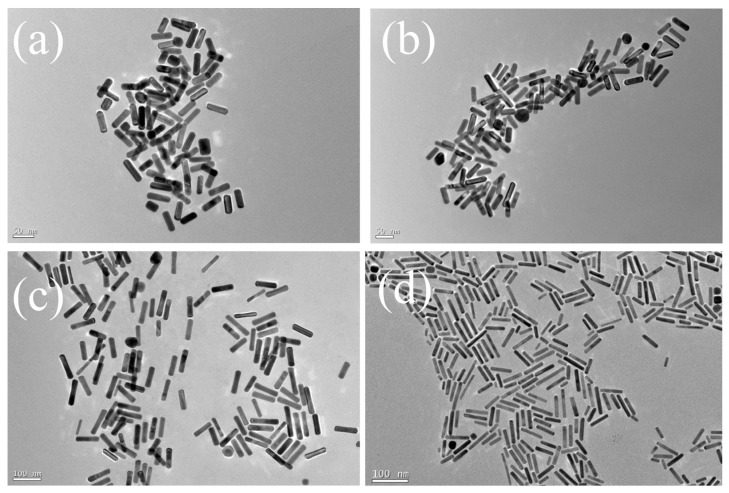
TEM images of GNRs with aspect ratios of (**a**) 3.39 (GNR-1), (**b**) 3.54 (GNR-2), (**c**) 4.19 (GNR-3), (**d**) 5.42 (GNR-4).

**Figure 2:**
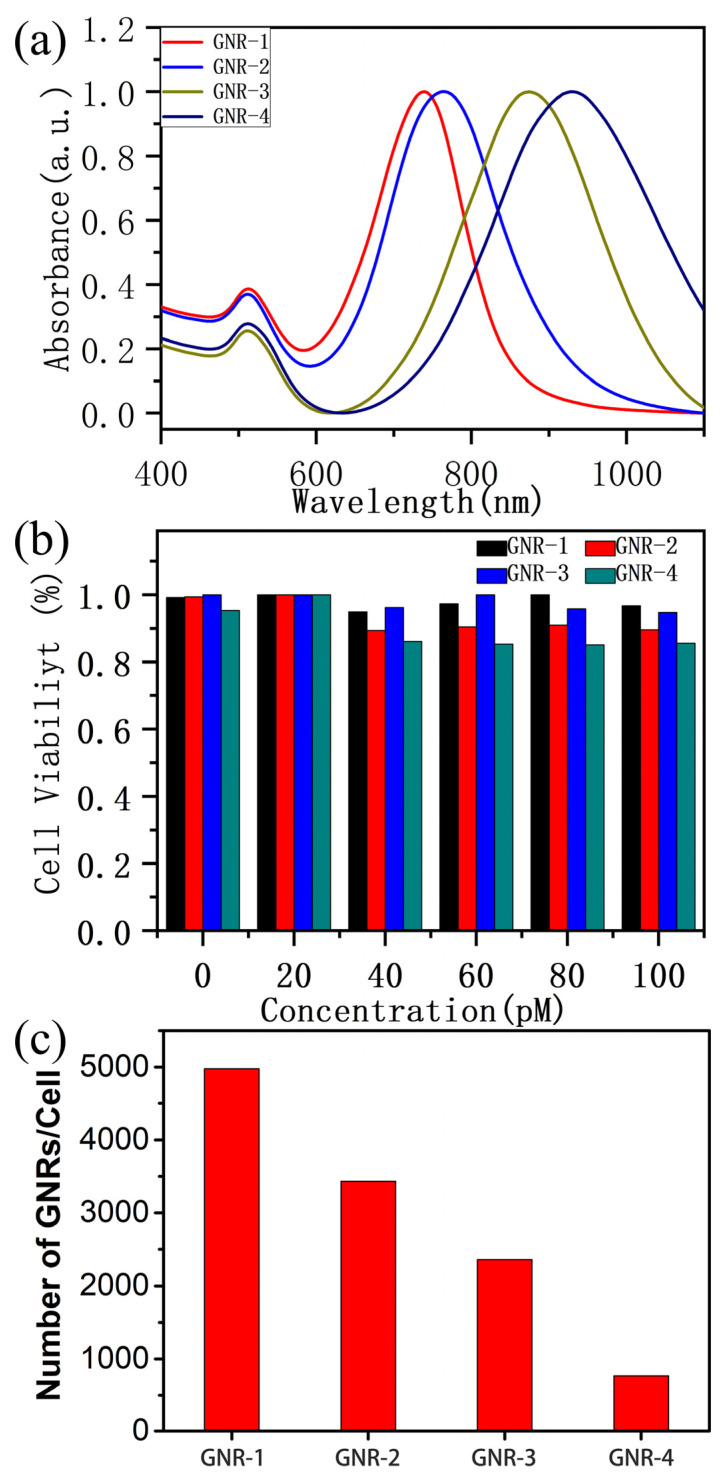
(**a**) The normalized UV-vis absorbance spectra of GNR-1, GNR-2, GNR-3, GNR-4. (**b**) Viability of HepG2 cells incubated with GNR-1, GNR-2, GNR-3, GNR-4 of different concentrations after 24 h. (**c**) The average amount of GNR-1, GNR-2, GNR-3, GNR-4 uptake by HepG2.

Four kinds of gold nanorods (GNRs) with different aspect ratios were synthesized by the seed-mediated growth method [38]. GNR particles with aspect ratios of 3.39 (GNR-1), 3.54 (GNR-2), 4.19 (GNR-3) and 5.42 (GNR-4) were obtained by reducing the amount of seeds. As shown in Figure 1, the lengths of GNR-1, GNR-2, GNR-3 and GNR-4 are 50.8 nm, 53.9 nm, 79.9 nm and 73.1 nm, respectively. The widths are 15.1 nm (GNR-1), 15.2 nm (GNR-2), 19.1 nm (GNR-3) and 13.4 nm (GNR-4). Figure S1 shows the size distributions of the GNRs. It could be seen that the size uniformity of GNR-3 and GNR-4 was lower than that of GNR-1 and GNR-2, which was probably caused by the strong reducibility of ascorbic acid, which increased the growth rate and the uneven distribution of Au seeds in the solution. The corresponding normalized UV-vis absorption spectra are shown in Figure 2a. The transverse surface plasma resonance (TSPR) peaks of GNRs are located at 520 nm. The longitudinal surface plasmon resonance (LSPR) peaks are 739 nm (GNR-1), 765 nm (GNR-2), 874 nm (GNR-3) and 930 nm (GNR-4), respectively. With the aspect ratios of GNRs increasing, there is a redshift for their LSPR peaks, which is consistent with previous reports [39].

### 2.2. Cytoxicity Studies and Cellular Uptake

Before studying the cytotoxicity of GNRs with different aspect ratios, it is necessary to consider that CTAB micelles are on the surface of GNRs. CTAB plays an important role in the morphologic formation of GNRs. On the surface of GNRs, CTAB loses Br- to form positively charged CTA+, which will destroy the function of the negatively charged membrane and lead to cell death [40,41]. Therefore, in order to determine the toxicity of GNRs with different aspect ratios more accurately, it was necessary to coat GNRs with PEG to reduce the biotoxicity caused by CTAB [42]. It is necessary to coat polyethylene glycol (PEG) outside to improve the biocompatibility. As shown in Figure S2, without coating PEG, HepG2 is incubated with GNR-CTAB for 24 h, and the shape of a large number of cells changes from spindle to circle, indicating that many cells die. On the contrary, there was no obvious change in cell morphology for HepG2 cells incubated with GNR-PEG for 24 h. The results show that coating with PEG could significantly reduce the biotoxicity of GNR-CTAB. In the following text, GNRs were coated with PEG by default.

After GNRs with different aspect ratios were cultured with the cells for 24 h, MTT (3-[4,5-dimethylthiazol-2-yl]-2,5 diphenyl tetrazolium bromide) assay was used to measure the cell viability. In Figure 2b, even the concentration of GNRs is as high as 100 pM (Au atom), and the cell viability is higher than 80% for all aspect ratio GNRs. These results indicate that the four GNRs had good biocompatibility.

**Figure 3:**
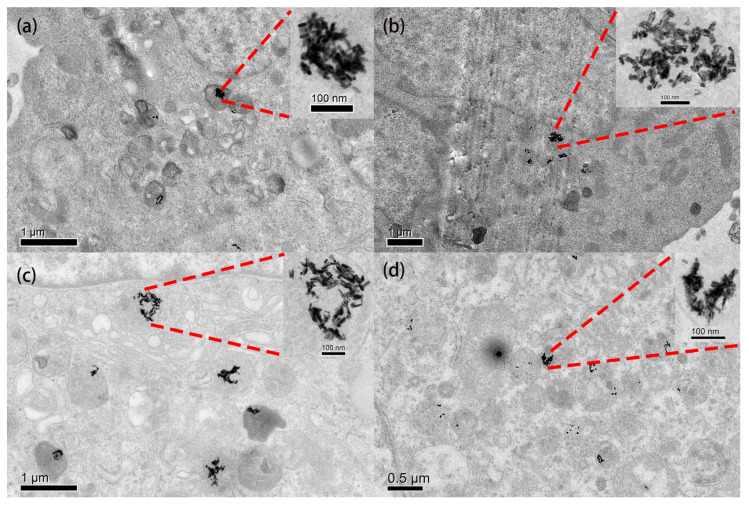
TEM images of GNR-loaded HepG2 cells (without fs laser irradiation). (**a**) GNR-1. (**b**) GNR-2. (**c**) GNR-3. (**d**) GNR-4. The inset images at the top right show a zoomed-in view of GNR clusters in cell vesicles.

The uptake of GNRs by HepG2 cells is investigated. Figure 2c shows the average uptake of different aspect ratio GNRs by single HepG2 cells. When the aspect ratio of GNRs increases, the amount of GNR uptake by single HepG2 cell decreases. Single HepG2 cells uptake about 4978, GNR-1, while for GNR-2, GNR-3 and GNR-4, single HepG2 cells uptake about 3432, 2358 and 768. It can be seen clearly that HepG2 prefers to uptake GNRs with short aspect ratios. This can also be verified by TEM images of HepG2 cells in Figure 3. As shown in Figure 3, GNRs accumulated in vesicles and lysosomes to form clusters. As for a single cluster, the number of GNRs in GNR-1 and GNR-2 is much larger than GNR-3 and GNR-4.

### 2.3. In Vitro Two-Photon Fluorescence Imaging

**Figure 4:**
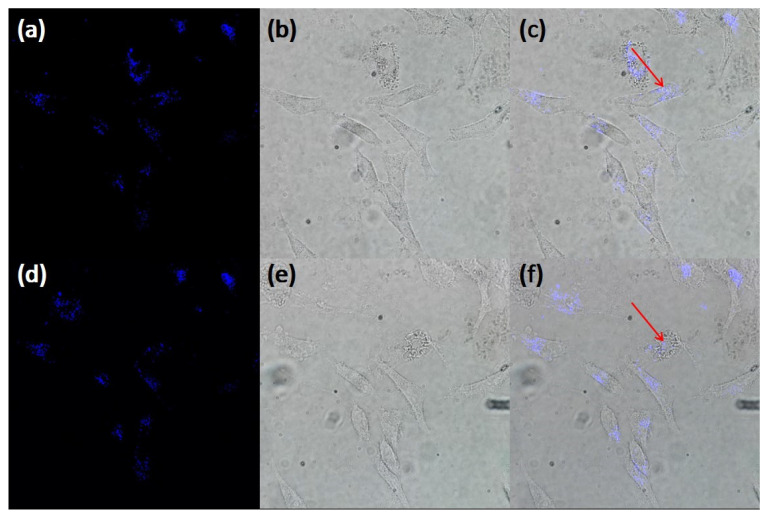
GNR-1 incubated with HepG2 cell 24 h, before photothermal therapy using 742 nm laser, (**a**) the two-photon fluorescence images and (**b**) bright-field images, (**c**) the overlap of (**a**,**b**). After photothermal therapy using 742 nm laser, (**d**) the two-photon fluorescence images and (**e**) bright-field images, (**f**) the overlap of (**d**,**e**). The red arrow points to the therapy site. The images are taken with 60× objective.

Upconversion fluorescence can be observed in GNRs under infrared laser excitation. Fluorescence can be used to locate the position of GNRs within cells, improving the efficiency of photothermal therapy. After 24 h of co-culture with GNRs, HepG2 cells are scanned by femtosecond laser and upconversion fluorescence. HepG2 cells are co-cultured with GNRs for 24 h and scanned by 742 nm (GNR-1), 776 nm (GNR-2), 874 nm (GNR-3) and 930 nm (GNR-4) lasers with power below the damage threshold, respectively. Figure 4a–c show the imaging before photothermal therapy, and Figure 4d–f show the imaging after photothermal therapy using a laser with power above the damage threshold. Figure 4a,d show the blue upconversion fluorescence of GNR-1 clusters in HepG2 cells excited by a 742 nm fs laser. Figure 4b,e are a bright-field image of cells. Figure 4c,f are obtained by overlapping Figure 4a,b. The blue and purple fluorescence in Figure 4c is all in the cell, indicating that GNRs-2 is taken up by HepG2 cells, and the location of GNRs-2 clusters in the cell can also be determined by using this fluorescence. Similar experimental results are also found in GNR-1, GNR-3 and GNR-4 experimental groups, as shown in Figures S3–S5.

### 2.4. In Vitro Photothermal Therapy

**Figure 5:**
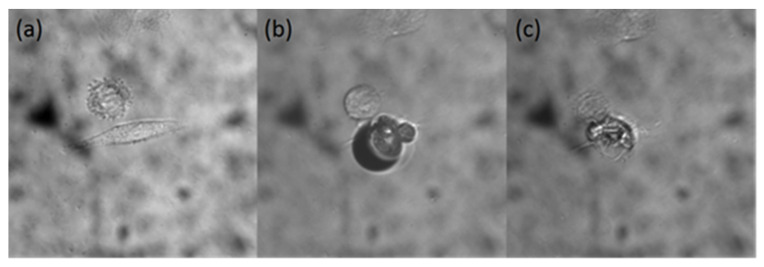
Using 742 nm laser to excite GNR-1 in HepG2 cell for photothermal therapy. (**a**) Before, (**b**) during, (**c**) after the therapy process. The images are taken with 60× objective.

After determining the position of GNR clusters using upconversion fluorescence, the cells are irradiated with laser power higher than the damage threshold for photothermal treatment. Figure 5a–c show the heating process of using a 742 nm fs pulse laser to excite GNR-1 in HepG2 cells. Before exciting, the shape of HepG2 is that of a spindle, as shown in Figure 5a. During the irradiation process with the femtosecond laser, the temperature near GNR clusters sharply increases. As shown in Figure 5b, it can be observed that bubbles appear near the laser irradiation point during laser treatment. In the exciting process, the shape of HepG2 cells changed from a spindle shape to irregular broken quasi-circles in Figure 5c. The same condition for GNR-2, GNR-3 and GNR-4 can be found in Figures S6–S8.

**Figure 6:**
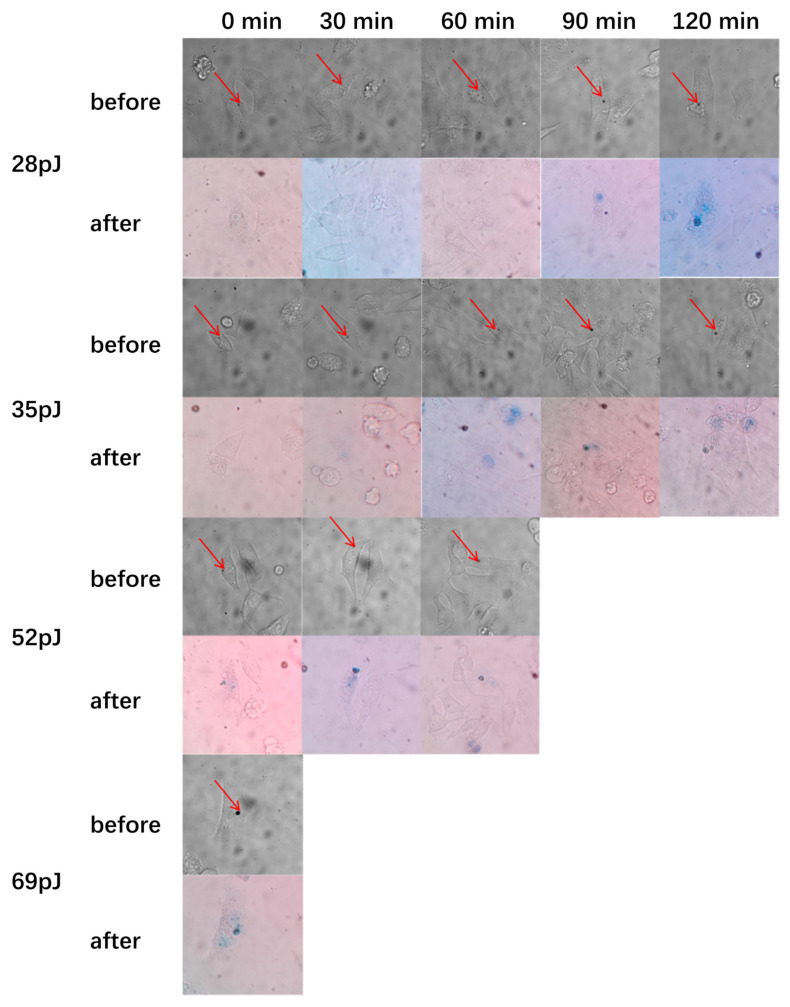
Photothermal treatment of GNR-1 under 742 nm fs pulsed laser. The laser focuses on the GNR-1 clusters in the HepG2 cells marked with red arrows. A series of fs laser energy 28 pJ, 35 pJ, 52 pJ, 69 pJ are set up to explore the relationship between time required for cell death and fs laser energy. Above and below images are cell state before and after photothermal treatment. The cells are stained with trypan blue dye at an interval of 30 min after photothermal treatment to observe whether the cells are dead. Blue inside cells indicates that the cells have died. The images are taken with 60× objective.

In order to avoid cell necrosis caused by high laser power and subsequent inflammation of nearby tissues, photothermal treatment with different laser power to induce cancer cell death has also been studied to achieve the purpose of inducing cell death with low power. After incubation of GNR-1 with HepG2 cells for 24 h, the cells are exposed to a focused 780 nm laser of different power for 20 s. Trypan blue dye is used to determine whether cells died. If the cells are dead, trypan blue dye could enter the nucleus and stain it blue. Figure 6 shows cell images before and after laser treatment. As can be seen from Figure 6, cells treated with a high-power laser die immediately, while those treated with a low-power laser die after a certain period of time. When the input laser power is 28 pJ (measured before entering the microscope), the cells die after being treated for 120 min. HepG2 cells die immediately after 20 s of irradiation with a laser pulse energy of 69 pJ. These results indicate that the time interval between photothermal treatment and cell death decreases with the increase in laser power.

After the cluster was focused by a laser, the medium temperature near the cluster increased. Cellular organelles are thermally damaged, leading to cell death. The low-power laser treatment prevents cell necrosis and reduces inflammation caused by cell necrosis.

In order to compare the photothermal efficacy of GNRs of different sizes, the experimental results of laser treatment of GNR-2, GNRs-3 and GNRs-4 are shown in Figures S9–S11. Among them, group GNR-2 uses a femtosecond laser of 776 nm wavelength, group GNR-3 uses a femtosecond laser of 862 nm and group GNR-4 uses a femtosecond laser of 920 nm. Table 1 summarizes the time for HepG2 death after laser treatment with different energy. The energies for immediately dying HepG2 are 69 pJ (GNR-1), 104 pJ (GNR-2), 138 pJ (GNR-3) and 138 pJ (GNR-4). It seems that GNRs with smaller aspect ratios require less pulse energy to induce HepG2 cell death. This is probably due to the fact that the uptake of GNRs with lower aspect ratios by HepG2 cells was very high, which can be verified in Figure 2c. Meanwhile, plasmon coupling and the “hot spot” where the energy is concentrated can be observed in multiple gold nanorod assembled clusters, so that the temperature could increase drastically. Figure S12 indicates that cells without co-cultivation with GNRs are irradiated by lasers of various wavelengths, with a pulse energy of 276 pJ for 20 s. It can be seen that the cells are not damaged.

## 3. Discussion

In order to analyze the photothermal performance of GNRs of different aspect ratios, a series of photothermal simulations was performed using COMSOL 6.0 software [5,6,7,34,43,44,45]. According to Figure 3, a theoretical model of the distribution of GNR clusters was built. The free electrons inside GNRs oscillate under the excitation of an incident laser. The distribution of the electromagnetic field in space obtained by the interaction between the electromagnetic field generated by the free electron oscillating and the electromagnetic field of the incident light obeys the Helmholtz equation [6,34,45]:

(1) $$\nabla \times \mu_{r}^{-1}\left(\nabla \times E\right)-k_{0}^{2}\left(\varepsilon_{r}-\frac{j\sigma }{\omega \varepsilon_{0}}\right)E=0$$

Through this, we obtained the electric field distribution E. In Equation (1), $\mu_{r}$ is the relative permeability, which, in this case, was 1, both for water and gold; $\varepsilon_{r}$ is the relative permittivity [6,34]; $k_{0}$ is wave number; ω is angular frequency; j is imaginary unit; and σ is conductivity. Since the imaginary part of permittivity of Au is not zero, when excited by a laser at the frequency of LSPR, upon surface plasmon formation, nonradiative relaxation occurs through electron–phonon and phonon–phonon coupling, efficiently generating localized heat that can be transferred to the surrounding environment. 

GNRs could serve as light-to-heat converters. The corresponding volumetric power density of heat generation can be written as [46,47,48]:

(2) $$qr=\frac{1}{2}\varepsilon_{0}\omega lm\left(\varepsilon_{r}\right){\left|E\right|}^{2}$$

qr is volumetric power density, $\varepsilon_{0}$ is permittivity of vacuum, $\varepsilon_{r}$ is relative dielectric constant and E is electric field intensity. The kinetic energy of a portion of free electrons of GNRs was increased by absorbing the energy of incident photons. These high-energy electrons were unstable in terms of energy level and exchanged energy between electrons whose kinetic energy was not high through electron–electron scattering in a very short time (usually within tens of femtoseconds) [49,50,51]. Then, energy exchange happened between electrons and the lattice, and the lattice temperature increased. As the lattice temperature within the entire GNR increased, the GNR also exchanged energy with the surrounding medium, resulting in an increase in the temperature of the medium. This process can be described by the double-temperature equation.

(3) $$S\left(t\right)=\frac{qr}{\sqrt{2\pi }t_{\sigma }}\exp\left(\frac{{-\left(t-t_{0}\right)}^{2}}{2t_{\sigma }^{2}}\right)$$

(4) $$C_{e}\frac{\partial T_{e}}{\partial t}=\nabla \cdot \left(k_{e}\nabla T_{e}\right)-g\left(T_{e}-T_{l}\right)+S\left(t\right)$$

(5) $$C_{l}\frac{\partial T_{l}}{\partial t}=\nabla \cdot \left(k_{l}\nabla T_{l}\right)+g\left(T_{e}-T_{l}\right)-G\left(T_{l}-T_{m}\right)\frac{S}{V}$$

(6) $$\begin{array}{c} \rho_{m}C_{m}\frac{\partial T_{m}}{\partial t}=\nabla \cdot \left(k_{m}\nabla T_{m}\right)\;+G\left(T_{l}-T_{m}\right)\frac{S}{V} \end{array}$$

Equation (3) represents the change in thermal power density with the laser pulse time, $t_{0}$ is the time the pulse peak appeared, $t_{\sigma }=t_{l}/(2\sqrt{\ln2})$, $t_{l}$ = 130 fs was the pulse width. Equations (4)–(6) represent the coupling relationship between the temperature of electron $T_{e}$, lattice $T_{l}$ and medium (due to the large proportion of water in the cell, the water parameter is substituted into the medium parameter). $T_{m}$. g was the coupling coefficient of energy transfer by electrons to the lattice, and G was the coupling coefficient of energy transfer by the lattice to water around GNRs. $C_{e}$, $C_{l}$ and $C_{m}$ were the heat capacity of the electron, lattice, water; $k_{e}$, $k_{l}$ and $k_{m}$ were the thermal conductivity of the electron, lattice, water; $\rho_{m}$ was the mass density of water. Their values can be found in a previous study [37] or the Supporting Information Table S1. In the simulation, the radius of the Gaussian pulse laser was 1000 nm, and the pulse frequency was 76 MHz. The energy of the laser for GNR-1, GNR-2, GNR-3 and GNR-4 was 28/3 pJ. Considering a laser propagated through a microscope could decrease, we multiplied 1/3 on the base of experiments in the simulation. 

**Figure 7:**
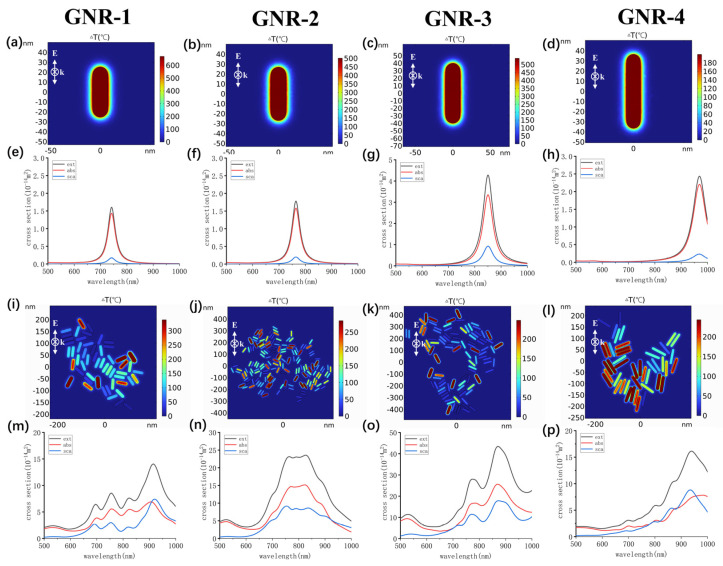
The wavelengths used in simulations were 742 nm (GNR-1), 776 nm (GNR-2), 862 nm (GNR-3), 920 nm (GNR-4). The maximum temperature increase in water when the laser irradiated single GNR (**a**–**d**) and GNR clusters (**i**–**l**). The absorption, scattering and extinction cross-sections of single GNR (**e**–**h**) and GNR clusters (**m**–**p**). The laser energy was 28/3 pJ in the simulation.

According to Equations (1)–(6), the rise in temperature and absorption cross-sections of individual GNRs and clusters was simulated and is shown in Figure 7. Figure 7 shows that the absorption peaks corresponding to a single GNR are 742 nm, 764 nm, 850 nm and 969 nm, which are very close to the experimental values of 739 nm, 765 nm, 874 nm and 930 nm. Due to production limitations, it is difficult to produce particles with very uniform sizes for GNR-4 with a large aspect ratio. Therefore, the absorption peak calculated based on the average particle size summarized from TEM images is different from the peak measured in actual experiments (39 nm). Figure 7a–d show the temperature rise in a single GNR excited by a vertically polarized laser. For GNR-1, it can reach 600 degrees, while GNR4 only has 180 degrees. It is possible that the experimental wavelength differs significantly from the theoretical absorption peak. According to the results of previous studies [47], at resonance, most of the heat originates from the center of the rod rather than from its extremities. This can be understood by the fact that the electronic current responsible for the Joule effect mostly flows in the center of the nanorod, while the extremities mainly accumulate charges, meaning that thermal and optical hot spots usually do not coincide. Figure S14 shows the internal field enhancement of a single GNR1. It reaches 18 at a vertical polarization laser incidence. However, for the horizontally polarized laser incidence, the internal field enhancement is 0.31, so the corresponding heating amplitude is only 0.16 degrees. For the GNR-1 cluster, the maximum internal field enhancement of the nanorod under a vertically polarized laser is 12 degrees, and the highest temperature rise point is 325 degrees. Under horizontal polarization, the maximum internal field enhancement of the nanorod is 11 degrees, and the highest temperature rise point is 230 degrees. These results indicate that the polarization direction of the laser has a significant impact on the heating of a single GNR. For GNR clusters, the polarization sensitivity decreases. Comparing Figure 7e,f,m–p, it can be seen that the cluster has a larger absorption cross-section and a wider response wavelength range than a single GNR. For example, GNR-2, the peak value of a single GNR absorption cross-section is 1.58 × 10^−14^ m^2^ (Figure 7f), with a half width at half height of about 50 nm. The absorption cross-section of the cluster is 15.16 × 10^−14^ m^2^, and the width is 195 nm. 

**Figure 8:**
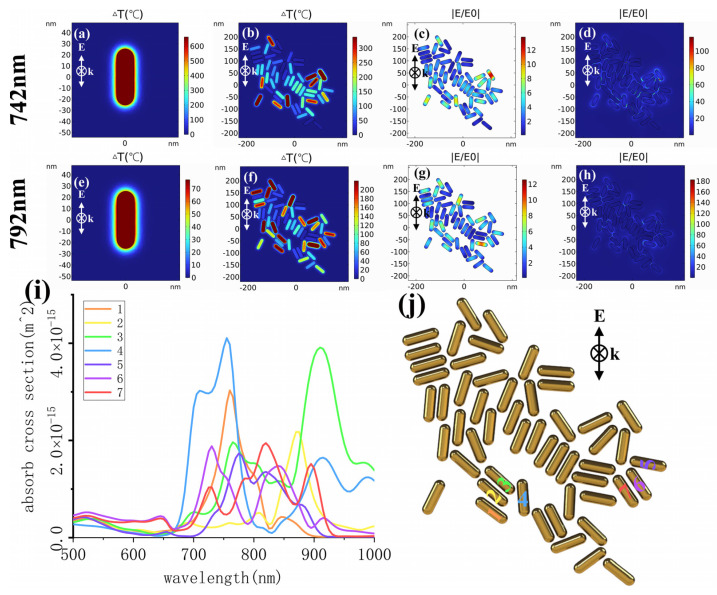
The photothermal response of single GNR-1 and GNRs-1 cluster at a wavelength of 742 nm (**a**–**d**) resonance and 792 nm (**e**–**h**) off-resonance. The maximum temperature increase in water on single GNR-1 (**a**,**e**) and GNRs-1 cluster (**b**,**c**). The inner electric field enhancement (**c**,**g**) and outer electric field enhancement (**d**,**h**) of GNRs-1 cluster. Absorption spectra (**i**) and locations (**j**) of seven GNRs in GNRs-1 cluster. The energy used in the simulation is 28/3 pJ.

After the formation of clusters, the wavelength response range of the GNR photothermal effect was expanded. In order to investigate this phenomenon in detail, taking GNR-1 as an example, the temperature rise after laser excitation at both resonant and off-resonant wavelengths for individual GNR particles and clusters was calculated (Figure 8). Figure 8a shows that a single GNR-1 heats up by 600 degrees when the excitation wavelength is 742 nm (GNR-1 LSPR peak). When excited by a laser with an off-resonant wavelength of 792 nm, the temperature rise is only 70 degrees. For clusters, the corresponding temperature rise for 742 nm is 325 degrees, and for 792 nm, it is 230 degrees. From Figure 8b,f, it can be seen that the particles heated up within the cluster are different for different excitation wavelengths. In order to investigate the causes of these differences, the internal and external field enhancement of GNR-1 clusters was calculated in both cases. It can be seen that external field enhancement induces an internal field enhancement in GNR, while internal field enhancement induces a temperature rise in GNR. The coupling between GNRs also affects the external field distribution of GNRs. GNRs within clusters interact with each other. As shown in Figure 8i, the absorption cross-sections of several representative GNRs as members of the cluster are calculated. The different absorption cross-section curves in Figure 8i correspond to particles labeled with different numbers in Figure 8j. From Figure 8i, it can be seen that the absorption peaks corresponding to different particles are distributed at different wavelength positions. These results in corresponding particles in the cluster show that they are able to achieve a higher temperature rise under laser excitation at different wavelengths. Therefore, the sensitivity of the cluster photothermal effect to the excitation laser wavelength is reduced. For the other GNRs, the pulse energy used in the experiment was substituted to calculate their rise in temperature and internal and external field enhancement under resonance or off-resonance wavelength laser excitation, and similar conclusions were obtained (Figure S15).

We conducted calculations, using GNR1 and GNR4 as representatives, to explore the photothermal conversion mechanism of different sizes of GNR after laser interaction. Figure 8, Figures S13 and S14, respectively, calculate the internal field enhancement and temperature rise of two types of GNRs under four conditions: resonance (vertical, horizontal polarization) and off-resonance (vertical, horizontal polarization). It can be seen that under vertical polarization conditions (including resonance and off-resonance), the internal length enhancement of a single GNR4 is greater than that of GNR1. Particularly, in the case of off-resonance, a single GNR4 can heat up by 200 degrees, but GNR1 is only 70 degrees. But, after forming clusters, the temperature rise in the two is very close. This is because small-sized GNRs are more compact in clusters and have more effective electromagnetic coupling. In the case of horizontal polarization, the heating amplitude of a single GNR4 is lower, and the temperature rise after forming clusters is slightly lower than that of GNR1 clusters in the case of off-resonance, indicating that GNR4 is more affected by laser polarization.

After GNR forms clusters in the cell, the restriction on the wavelength and polarization direction of the light source is reduced, which is conducive to the flexible use of different light sources in practical operation and is conducive to the practical application of photothermal therapy. For practical applications, small-sized GNRs have greater advantages among the four types of GNRs. The reason is as follows: 1: Cells have a higher uptake of small-sized GNRs. 2: Due to its morphological limitations, the formation of clusters of large-sized GNRs is not as compact as small-sized GNRs, resulting in lower electromagnetic coupling efficiency compared to small-sized GNRs. 3: The absorption band of GNR clusters shows a redshift compared to a single GNR absorption peak. The absorption peak of large-sized GNR clusters is likely to exceed the first biological window (about 700 nm–950 nm).

## 4. Materials and Methods

### 4.1. GNR Synthesis of Different Aspect Ratios

Auric acid (HAuCl_4_·3H_2_O), trisodium citrate (C_6_H_5_O_7_Na), ascorbic acid (C_6_H_8_O_6_) and Hexadecyl trimethyl ammonium bromide (CTAB) were purchased from aladdin. Sodium borohydride (NaBH_4_) was purchased from Tianjin Damao Chemical Reagent Factory. Polyethylene Glycol (HS-PEG1500-SH) was purchased from Sigma-Aldrich (Shanghai, China). Digital heating circulating water bath was purchased from Gongyi Yuhua Instrument company (Gongyi, China).

The synthesis of GNRs is based on Murphy’s work [1] with minor modifications. Briefly, the citric-acid-coated seeds were first synthesized by mixing HAuCI4 (10 mL, 0.5 mM) and trisodium citrate (10 mL, 0.5 mM), adding cold NaBH4 solution (0.6 mL, 100 mM) under intense agitation, and then standing at 30 °C for 3 h to obtain gold seeds. In the second step, gold nanorods with different aspect ratios were grown using gold seeds. HAuCI4 (20 mL, 0.25 mM) and CTAB (20 mL, 100 mM) were mixed, ascorbic acid (0.25 mL, 0.1 M) was added and stirred until the solution was clarified, and then gold nanorods were obtained by adding gold seed solution and growing at 30 °C for 12 h. The aspect ratio of gold nanorods changed according to the amount of gold seed added. The synthesized gold nanorods were dispersed into 1% PEG solution and stirred overnight to obtain PEG-coated gold nanorods.

### 4.2. Cell Culture and Cytotoxicity Experiments

HepG2 cell suspension and 96-well plate were prepared. HepG2 cell suspension was added into the 96-well plate with 100 μL per hole, and 100 μL PBS phosphate buffer was added into the outermost hole of the 96-well plate; after being cultured in the incubator for 12 h, gold nanorods with different concentrations of 2 nM and 4 nM were added to the experimental group. Then, 6 nM, 8 nM, 10 nM and control group (only the same amount of cell suspension was added) and blank group (only the same amount of culture medium was added) were set. Further, 20 μL 5 mg/mL 3-(4,5-dimethylthiazol-2-yl)-2,5-diphenyltetrazolium bromide (MTT) solution [2] was added to each hole containing cells and incubated for 4 h in an incubator (37 °C, 5% CO_2_); then, 150 μL DMSO was added after the supernatant was sucked out with pipette. Then, we connected the microplate reader (BioRad, Hercules, CA, USA) to the computer, selected a wavelength of 490 nm and vibration time of 600 s, put the 96-well plate on the designated position of the enzyme marker and obtained and recorded the light absorption value (OD value) of each hole.

In order to eliminate experimental errors, the experimental group, the control group and the blank group were set up. The experimental group added drugs into the cell suspension, the control group only contained cell suspension and the blank group only contained culture medium. The samples of the three groups need to be added with the same amount of MTT solution and DMSO. The cell survival rate was calculated using the following formula:

(7) $$Cell\;viability=\frac{{OD}_{experimental}-{OD}_{blank}}{{OD}_{control}-{OD}_{blank}}$$

### 4.3. Photothermal Therapy Experiments

The experimental device used in the photothermal therapy experiment is shown in Figure S15. For photothermal therapy experiments, a laser between is emitted from Ti/sapphire oscillator (Mira 900 S, Coherent, Santa Clara, CA, USA), filtered through bandpass filter to obtain 742 nm laser. Then, we guided 742 nm laser into the microscope (Axio Observer A1, Zeiss, Santa Clara, CA, USA) and focused the laser to irradiate GNR clusters in HepG2 cells. The cell state was observed in real time by an optical spectrometer (ANDOR, SR-500i-B1, Belfast, Northern Ireland) and a CCD camera (IMG, SC2000C, Shenzhen, China)

### 4.4. Cellular Uptake of GNRs

Five culture bottles were taken, HepG2 cells were inoculated in each culture bottle and gold nanorod solution with the same concentration and different aspect ratios was added to four of them, respectively; one was left as a blank group. After culture in the incubator for 24 h, the liquid in the culture bottle was removed with a pipette, 1 mL pancreatic enzyme was added to the cells for debonding treatment, and then the cell suspension was diluted; the cells were counted with a counting board, and the number of cells in the 1 mL cell suspension was calculated by cell count.

After absorbing 1 mL of the above counted cell suspension, adding 2 mL concentrated nitric acid and 0.5 mL concentrated hydrochloric acid and heating the boiling water bath to 60 °C, the acid was evaporated until the sample solution was clear and transparent. If the solution remains clear and transparent during the process, a small amount of deionized water can be added, and, finally, the volume of the solution will be roughly 0.2 mL. Further, 9.8 mL deionized water can then be added. The volume is set to 10 mL, and, finally, the gold content in the solution is determined by ICP-MS; then, the gold content in the individual cells is calculated.

### 4.5. Simulation

Figure S16 shows the geometry of the model. The GNR cluster was placed in a water sphere with 1.4 μm diameter, surrounded by a 300 nm thick perfect matching layer (PML) and 300 nm thick infinite source domain (IE). The perfect matching layer absorbed the light to approximate GNRs was in the infinite water, and the infinite source domain simulated the heat distribution in the infinite space. The colored arrows arranged along the *Z* axis represent the propagation direction along *Z* axis and polarization of the incident Gaussian laser along *Y* axis, while the XOY section represents the distribution of the temperature increase in water.

The wave optical module and partial differential equations module of COMSOL 6.1 software (http://cn.comsol.com/, accessed on 3 January 2024) were used to simulate the pulsed photothermal effect of GNRs. The calculation was divided into two steps. In the first step, the background field in the wave optical module was used for incident Gaussian light on the GNR cluster. The distribution of electric field in space was calculated using frequency domain solver, and then the thermal power density of the GNRs was obtained according to Equation (2). In the second step, the double-temperature Equations (4)–(6) were entered into the partial differential equation module. The change in temperature in space with time was obtained using transient solver. The specific parameters inputted into software are summarized in a table in Supporting Information.

## 5. Conclusions

In conclusion, the size dependence of gold nanorods for efficient and rapid photothermal therapy was studied to optimize treatment effects in this study. For practical applications, small-sized GNRs have greater advantages among the four types of GNRs: 1. More easily taken up by cells, the uptakes of GNR-1, GNR-2, GNR-3 and GNR-4 by single HepG2 cells were 4978, 3432, 2358 and 768. 2. After ingesting short gold rods, the laser energy required to kill cancer cells was lower, and the cell death time was shorter for short aspect ratios. For GNR-1 and GNR-2, the energies required for cell death in 90 min were 28 pJ and 52 pJ, while for GNR-3 and GNR-4, the energy required was 69 pJ. 3: Through simulation analysis, it was found that the aggregation of GNRs in cell vesicles led to a broadening of the absorption spectrum and weakening of the polarization sensitivity, which is conducive to the flexible use of different light sources in practical operation. Due to its morphological limitations, the formation of clusters of large-sized GNRs is not as compact as for small-sized GNRs, resulting in lower electromagnetic coupling efficiency compared to small-sized GNRs.

## Figures and Tables

**Table 1:** The time for HepG2 cell death irradiated by different energy fs laser after incubation with different GNRs.

		Energy	28 pJ	35 pJ	52 pJ	69 pJ	104 pJ	138 pJ
		
Aspect Ratio		Death Time						
GNR-1			90 min	60 min	30 min	0 min		
GNR-2				120 min	90 min	30 min	0 min	
GNR-3						90 min	30 min	0 min
GNR-4					120 min	90 min	30 min	0 min

## Data Availability

All data associated with this article are included in the article.

## Supplementary Materials

The supporting information can be downloaded at: https://www.mdpi.com/article/10.3390/ijms25042018/s1, references [52,53] are cited in Supplementary Materials.
