# Supplementary material for: Size Dependence of Gold Nanorods for Efficient and Rapid Photothermal Therapy
Source: Int J Mol Sci. 2024 Feb 7;25(4):2018. doi: 10.3390/ijms25042018 (PMC10888739; doi:10.3390/ijms25042018)
Supplement: Supplementary file 1 [file ijms-25-02018-s001.zip › ijms-2834579-supplementary.pdf]

Supporting Information

Size-dependence of Gold Nanorods for Efficient and Rapid Photothermal Therapy

Wei Zhou [1,2], Yanhua Yao[1], Hailing Qin[1], Xiaobo Xing [2], Min Ouyang[1,*], and Haihua Fan[1,*]

[1] Guangdong Provincial Key Laboratory of Nanophotonic Functional Materials and Devices, School of Information and Optoelectronic Science and Engineering, Guangdong Basic Research Center of Excellence for Structure and Fundamental Interactions of Matter, South China Normal University, Guang-zhou 510006, China; 2023010310@m.scnu.edu.cn(WZ); 1342691482@qq.com(YY); 1392124754@ qq.com(HQ); minoy@scnu.edu.cn(MO); fanhh@scnu.edu.cn(HF)

[2] Technology & Centre for Optical and Electromagnetic Research, South China Academy of Advanced Optoelectronics and National Center for International Research on Green Optoelectronics, South China Normal University, Guangzhou 510006, P. R. China; 2023010310@m.scnu.edu.cn(WZ);xingxiaobo@scnu.edu.cn(XX)

* Correspondence: fanhh@scnu.edu.cn; minoy@scnu.edu.cn

1. Materials and Methods

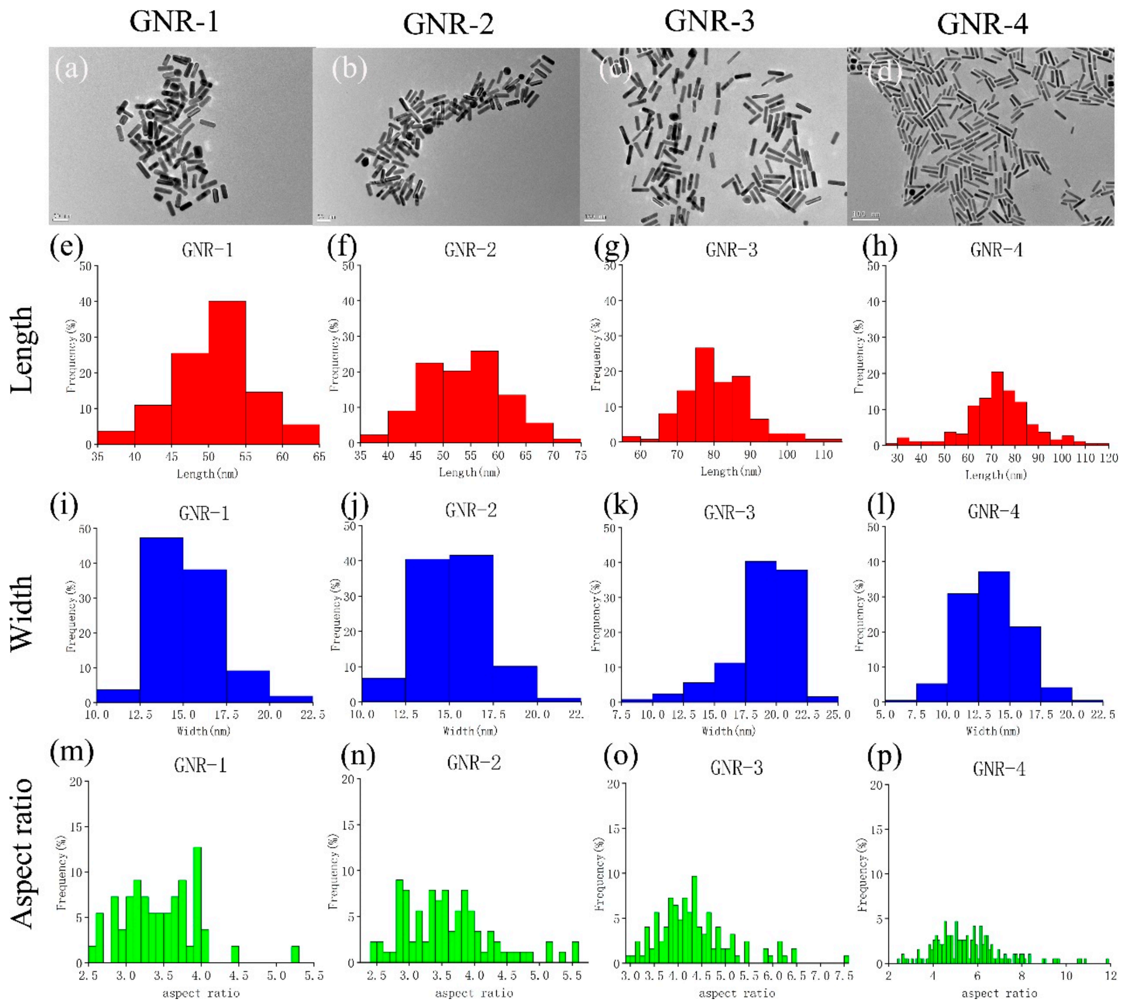

Figure S1. Size distributions of GNR-1 to GNR-4. (a-d) the TEM of GNR-1 to GNR-4. (e-h) the length distributions of GNR-1 to GNR-4. (i-l) the width distributions of GNR-1 to GNR-4. (m-p)

the aspect ratio distributions of GNR-1 to GNR-4.

2. Photothermal Experimental Data

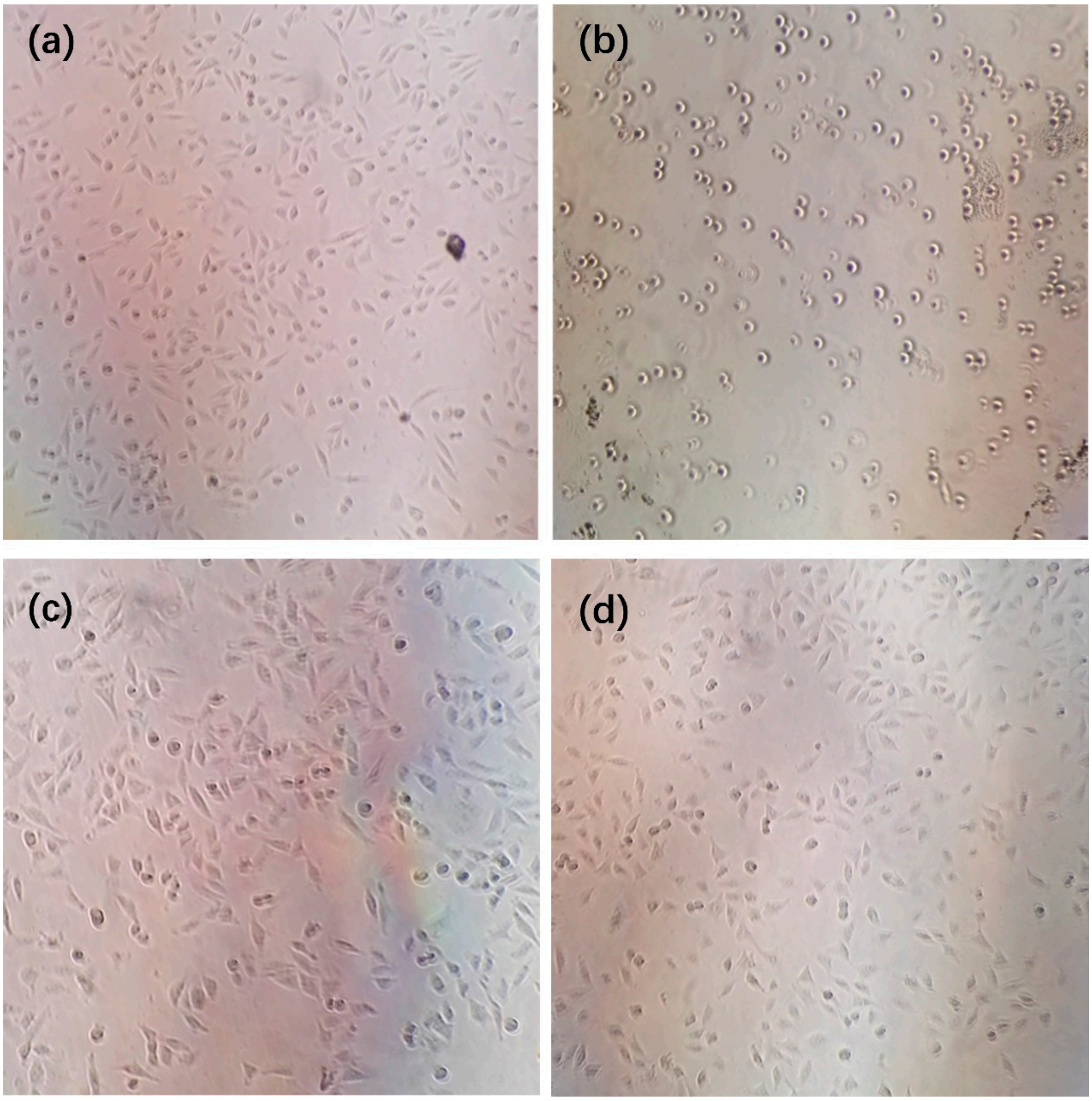

Figure S2. The images of HepG2 incubated with GNRs-CTAB 24h before **(a)** and after **(b)**, with GNRs-PEG 24h before **(c)** and after **(d)**.

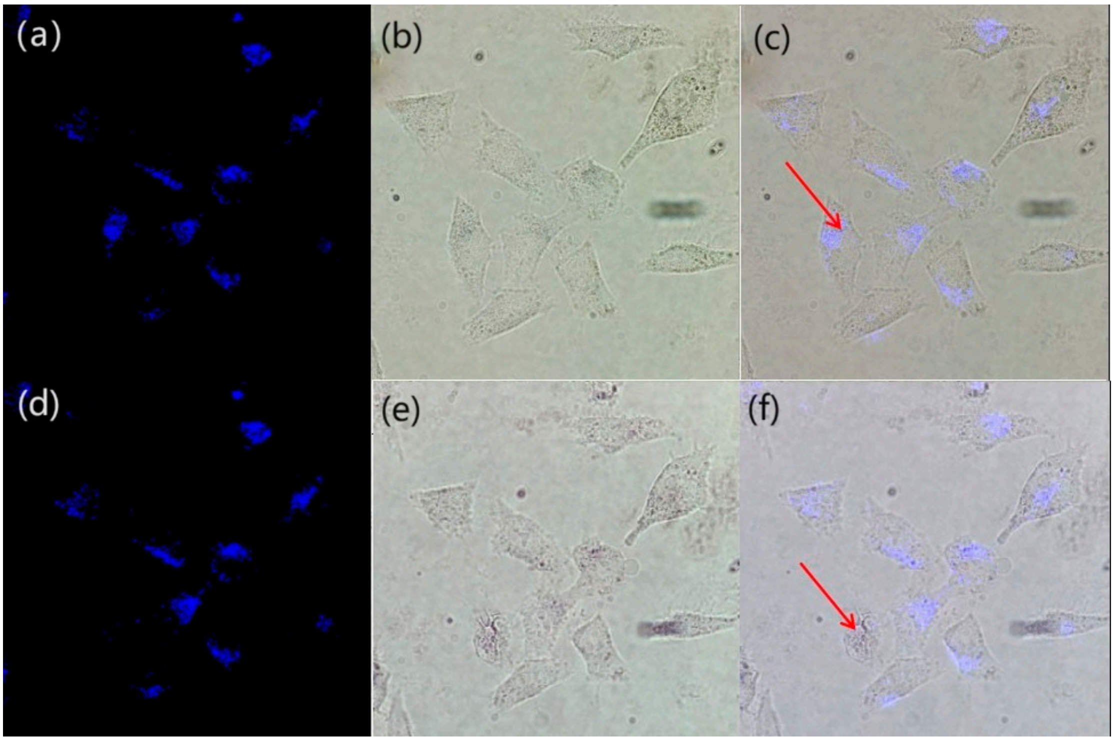

Figure S3. GNR-2incubated with HepG2 cell 24h, before photothermal therapy using 776nm laser, **(a)** the two-photon fluorescence images and **(b)** bright field images, **(c)** the overlap of (a) and (b). After photothermal therapy using 742nm laser, **(d)** the two-photon fluorescence images and **(e)** bright field images, **(f)** the overlap of (d) and (e). the red arrow point the therapy site.

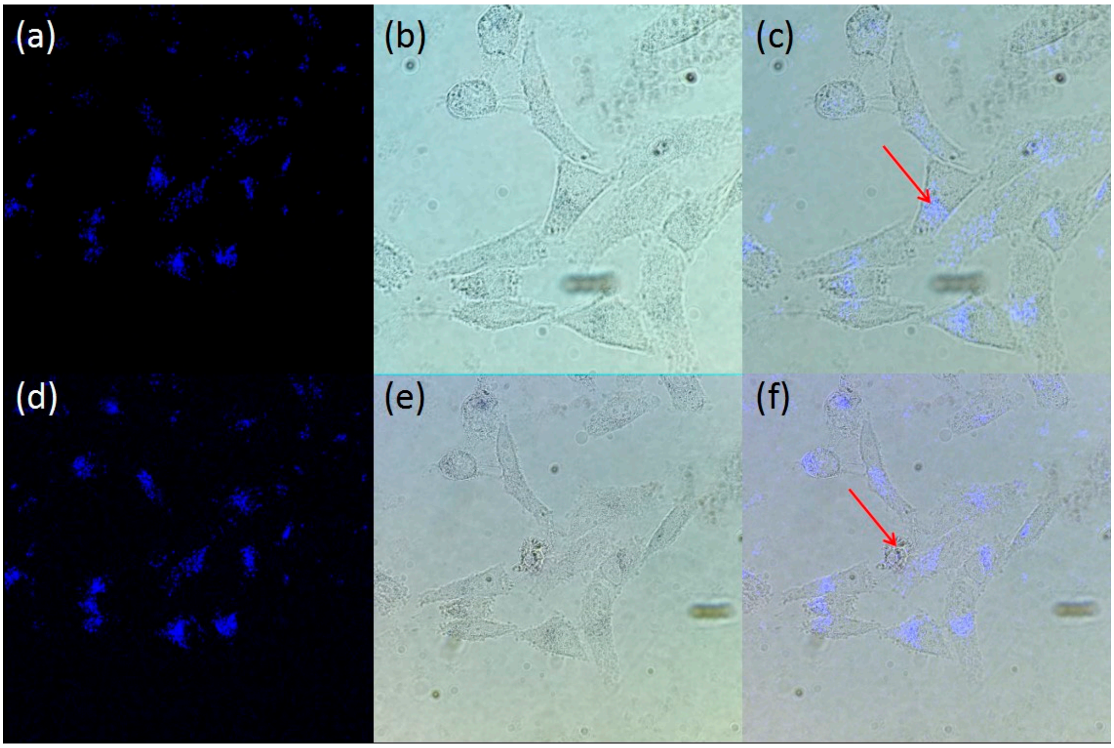

Figure S4. GNR-3 incubated with HepG2 cell 24h, before photothermal therapy using 862nm laser, **(a)** the two-photon fluorescence images and **(b)** bright field images, **(c)** the overlap of (a) and (b). After photothermal therapy using 862nm laser, **(d)** the two-photon fluorescence images and **(e)** bright field images, **(f)** the overlap of (d) and (e). the red arrow point the therapy site.

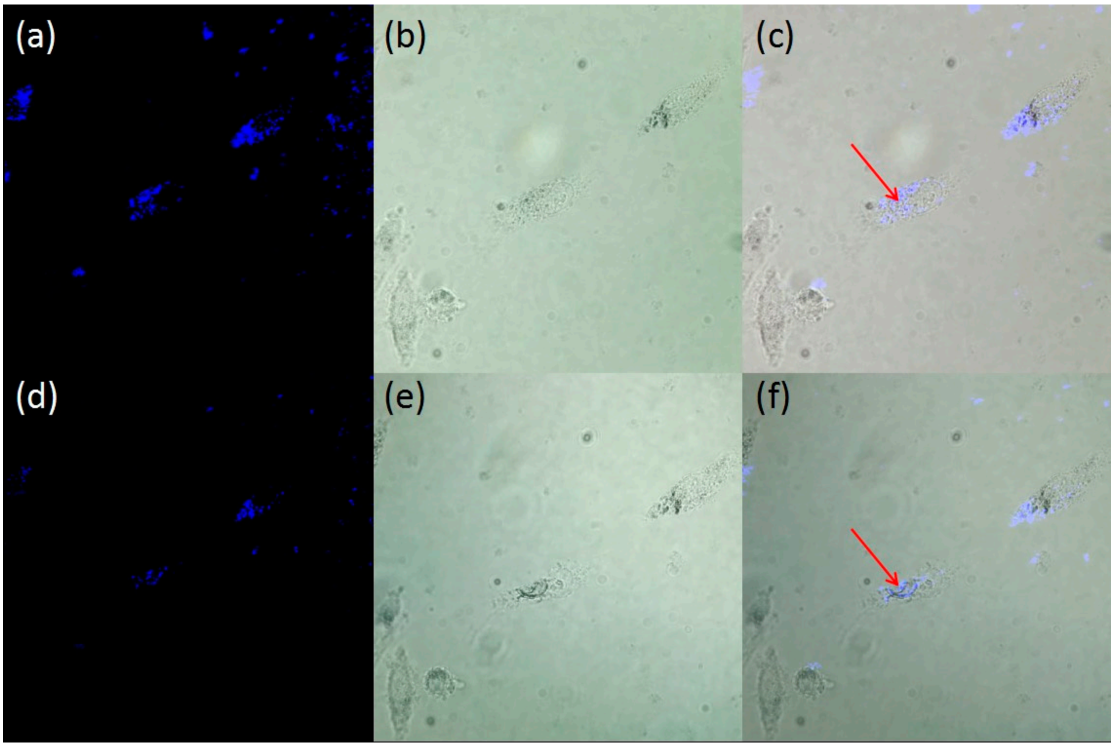

Figure S5. GNR-4 incubated with HepG2 cell 24h, before photothermal therapy using 920nm laser, **(a)** the two-photon fluorescence images and **(b)** bright field images, **(c)** the overlap of (a) and (b). After photothermal therapy using 920nm laser, **(d)** the two-photon fluorescence images and **(e)** bright field images, **(f)** the overlap of (d) and (e). the red arrow point the therapy site.

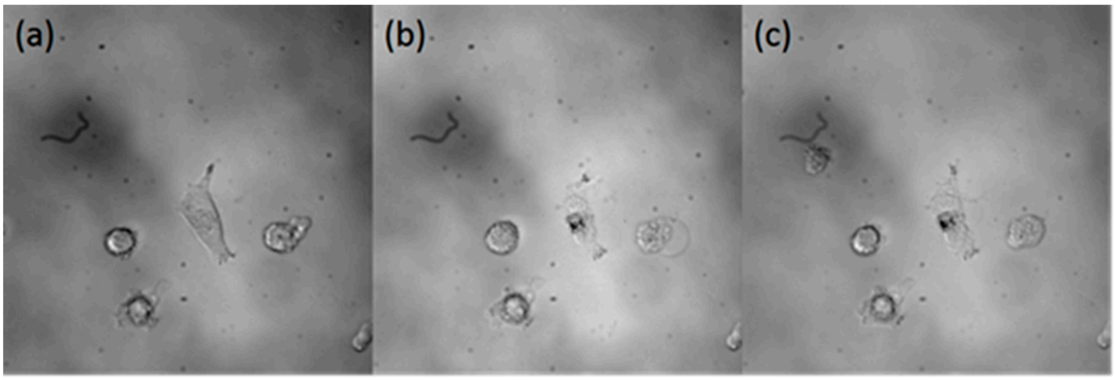

Figure S6. Using 776nm laser to excite GNR-2 in HepG2 cell for photothermal therapy. **(a)** before, **(b)** during, **(c)** after the therapy process.

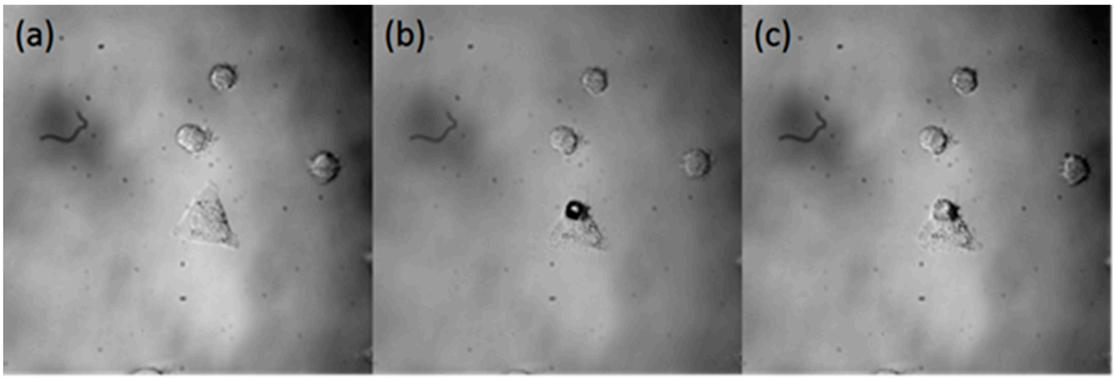

Figure S7. Using 862nm laser to excite GNR-3 in HepG2 cell for photothermal therapy. **(a)** before, **(b)** during, **(c)** after the therapy process.

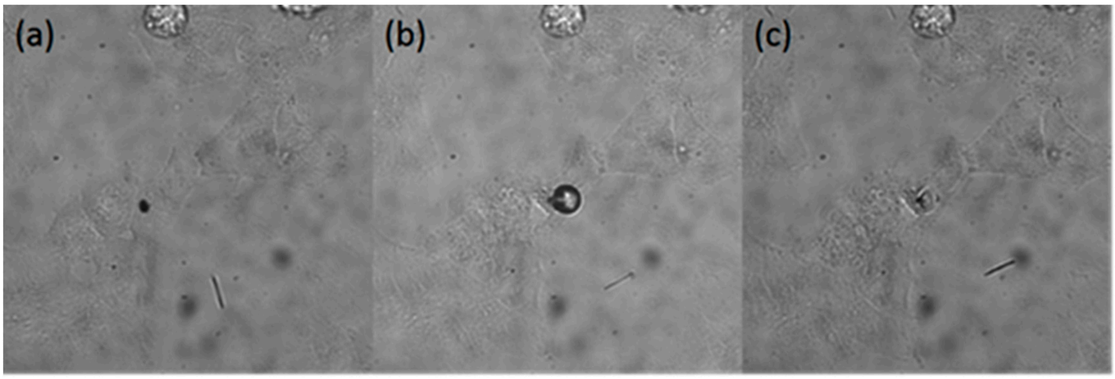

Figure S8. Using 920nm laser to excite GNR-4 in HepG2 cell for photothermal therapy. **(a)** before, **(b)** during, **(c)** after the therapy process.

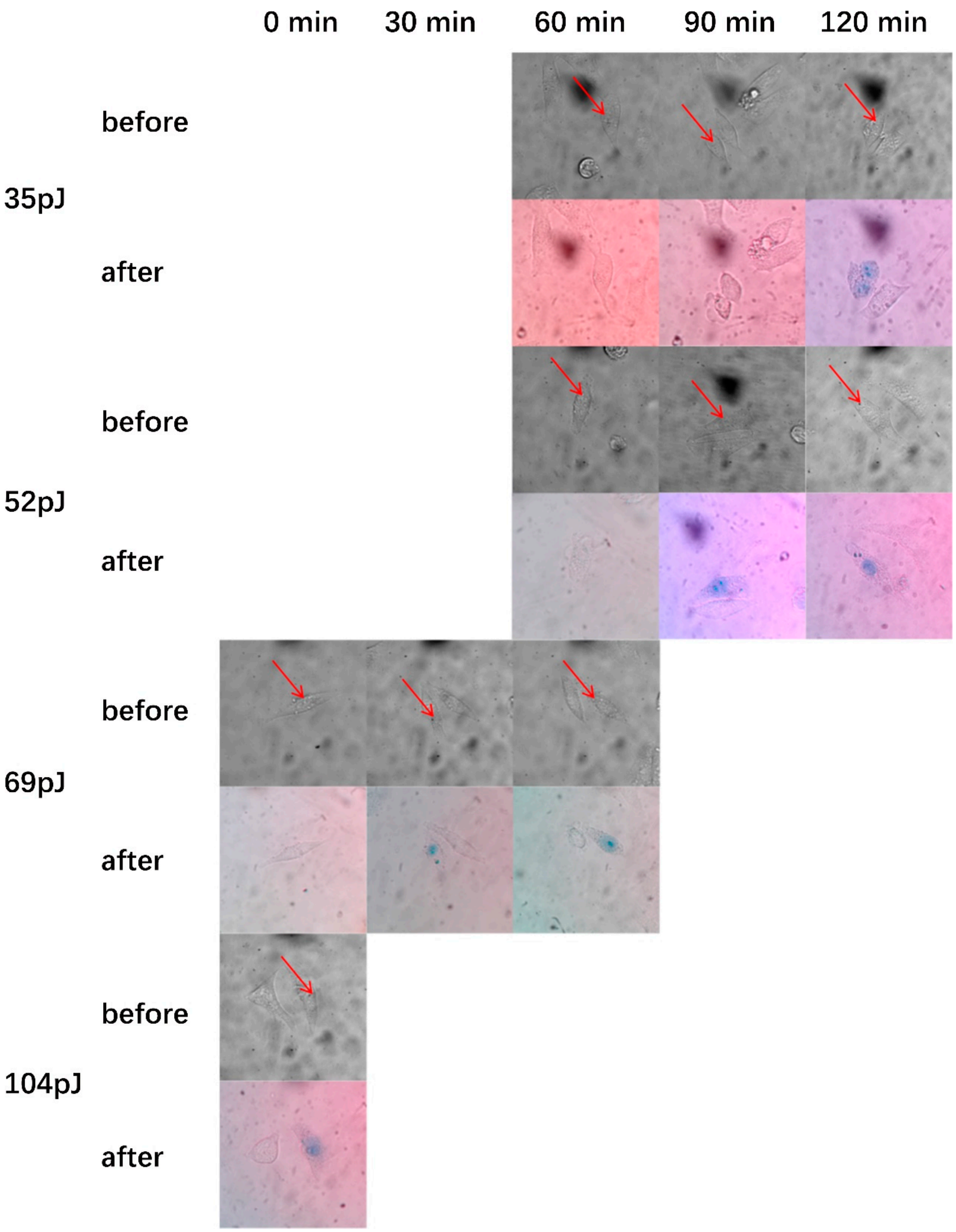

Figure S9. HepG2 incubated with GNR-2 for 24h，the bright field images before and after photothermal therapy. In the experiment, a focused 776nm laser with pulse frequency of 76MHz, pulse width of 130fs, the energy of 28pJ，35pJ, 52pJ, 64pJ irradiated the GNR-3 clusters which were indicated by red arrows in HepG2 cells, and then dyed by trypan blue Within 30 minute intervals.

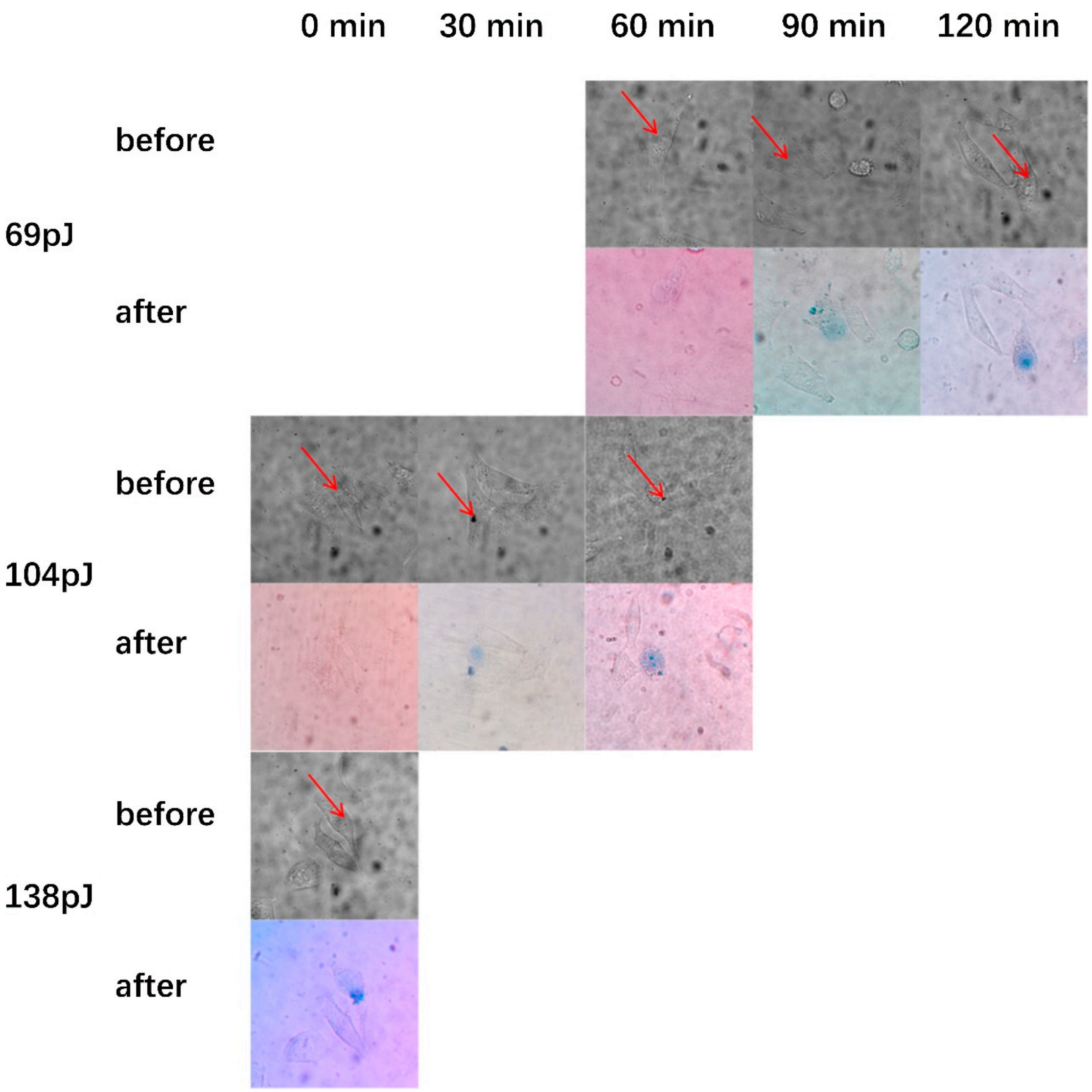

Figure S10. HepG2 incubated with GNR-3 for 24h, the bright field images before and after photothermal therapy. In the experiment, a focused 862nm laser with pulse frequency of 76MHz, pulse width of 130fs, the energy of 28pJ, 35pJ, 52pJ, 64pJ irradiated the GNR-3 clusters which were indicated by red arrows in HepG2 cells, and then dyed by trypan blue Within 30 minute intervals.

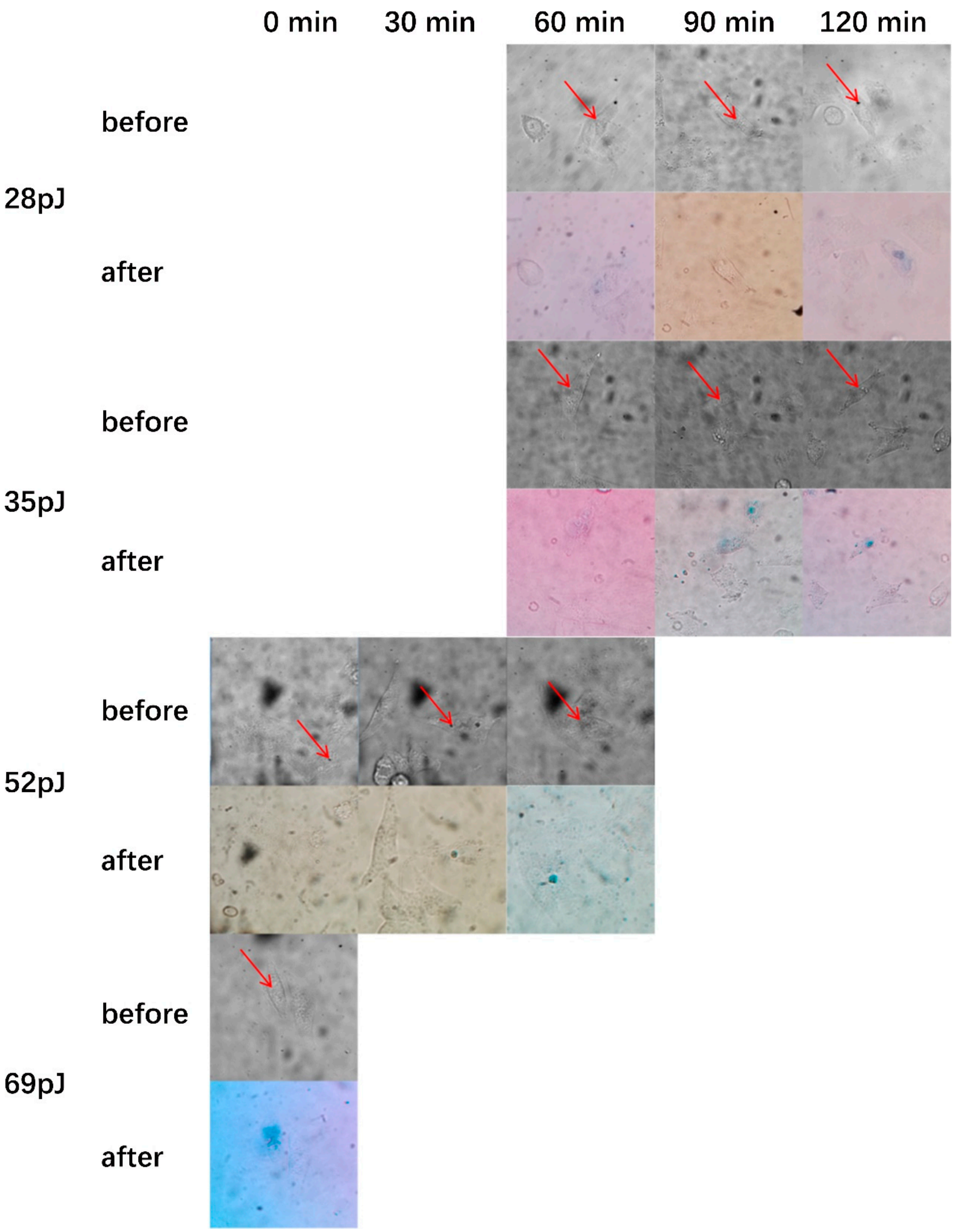

Figure S11. HepG2 incubated with GNR-4 for 24h， the bright field images before and after photothermal therapy. In the experiment, a focused 920nm laser with pulse frequency of 76MHz, pulse width of 130fs, the energy of 28pJ， 35pJ, 52pJ, 64pJ irradiated the GNR-3 clusters which were indicated by red arrows in HepG2 cells, and then dyed by trypan blue Within 30 minute intervals.

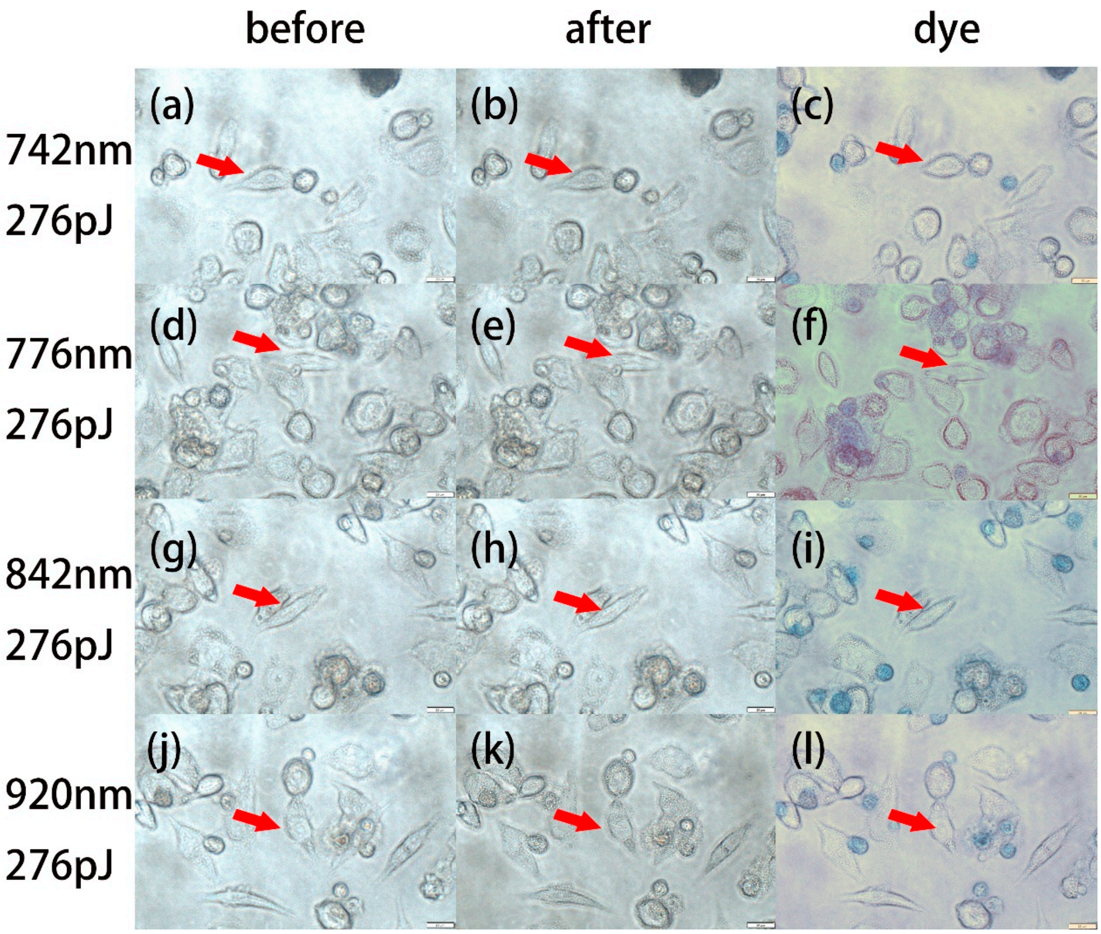

Figure S12. The image of HepG2 cells without GNRs after being irradiated by fs laser. The red arrows mark the cells underwent photothermal therapy.

3. Numerical Simulation

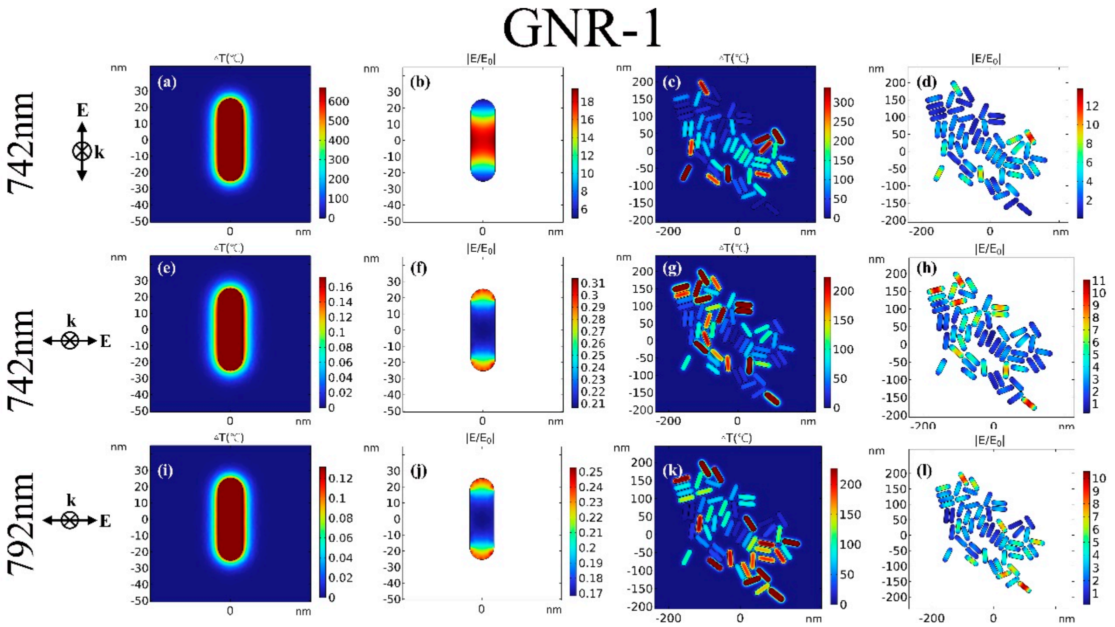

Figure S13. Effect of polarization and wavelength on photothermal response of single GNR-1 and GNRs-1 cluster. **(a-d)** the single GNR-4 and GNRs-4 clusters irradiated by on-resonant wavelength of 742nm laser with longitudinal polarization. **(e-h)** the single GNR-4 and GNRs-4 clusters irradiated by on-resonant wavelength of 742nm laser with transverse polarization. **(i-l)** the single GNR-4 and GNRs-4 clusters irradiated by off-resonant wavelength of 792nm laser with transverse polarization. ∣E/E0∣ indicated the outer or inner electric field enhancement, △T indicated the maximum temperature increasement of water.

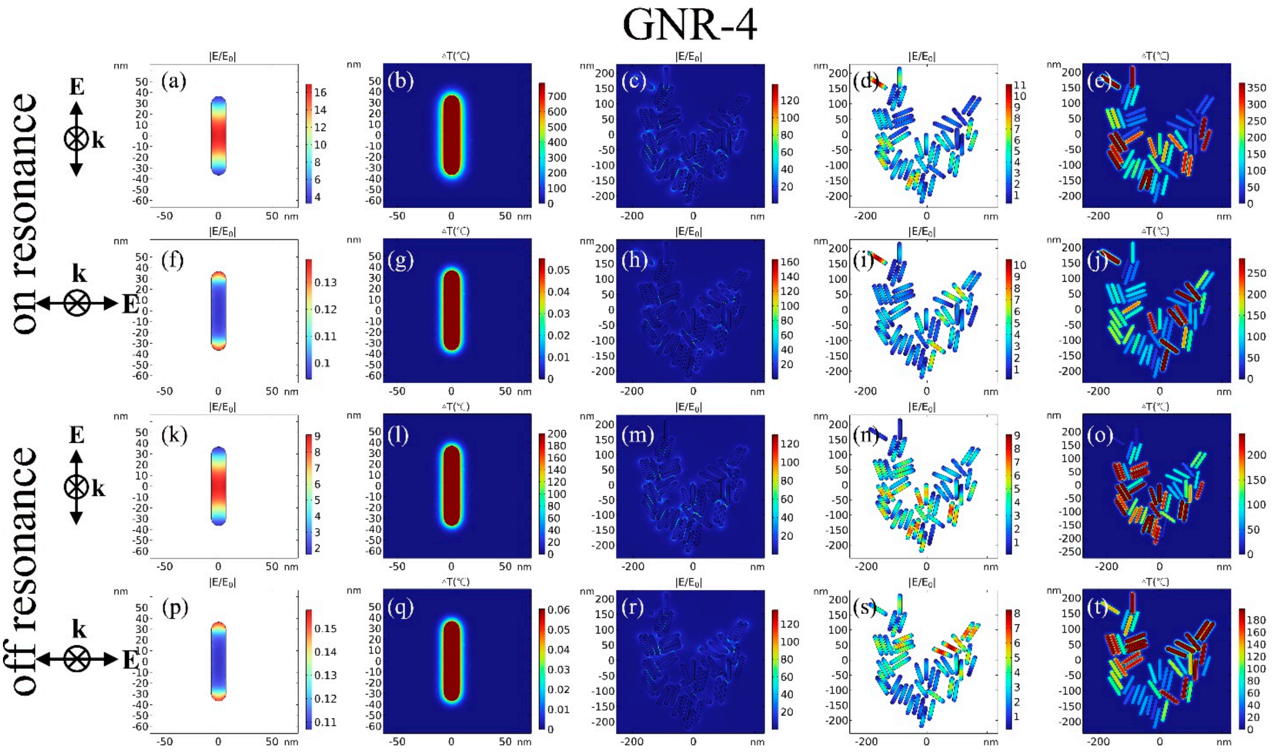

Figure S14. Effect of polarization and wavelength on photothermal response of single GNR-4 and GNRs-4 cluster. (a-j) the single GNR-4 and GNRs-4 clusters irradiated by resonant wavelength of 969nm. (k-t)

the single GNR-4 and GNRs-4 clusters irradiated by resonant wavelength of 920nm. ∣E/E0∣ indicated the outer or inner electric field enhancement, △T indicated the maximum temperature increasement of water

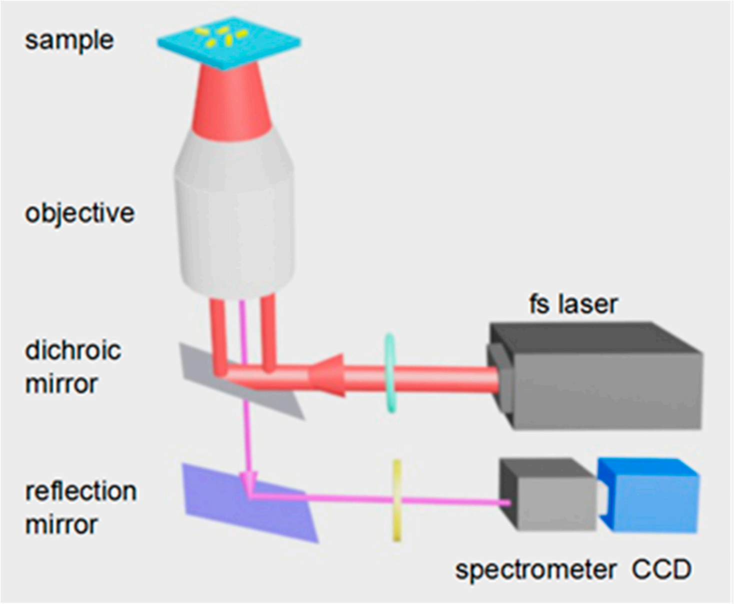

Figure S15. The experimental setup used in the photothermal therapy experiment.

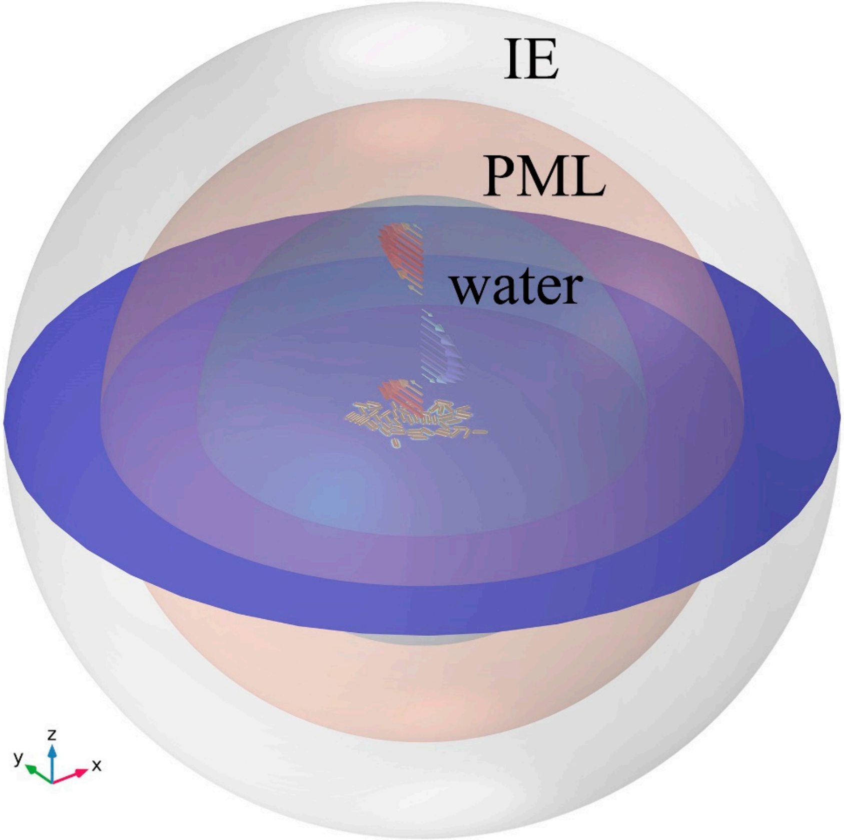

Figure S16. Geometric diagram of COMSOL in simulation.

Table S1. The parameters used in simulation

Parameter	Physical quantity	Value
μ_r	relative permeability	1
k_0	Wave number	2*pi/λ_0
λ_0	Wavelength	See article for specific values
ε_r	relative dielectric constant	For water,1.33.<br>For Au, Johnson and Christy [3].
ω	angular frequency	2*pi*c/λ_0, c is the speed of light 2.9979E8 m/s.
σ	conductivity	0
ε_0	permittivity of vacuum	8.8542E-12 F/m
t_0	the time of pulse peak appers	500 fs
t_σ	The standard deviation of a Gaussian function	$t_l/(2\sqrt{\ln 2})$, t_l is pulse width 130 fs.
C_e	The electron heat capacity	70 J/(m^3*K^2) *T_e
k_e	Thermal conductivity of electron	300 W/(m*K)
g	Electron-lattice coupling coecient	2*10^16 W/(m^3*K)
C_l	The lattice heat capacity	3*10^6 J/(m^3*K)
k_l	Thermal conductivity of lattice	0.001*k_e
G	Thermal conductivity at the GNR/water interface	105*10^6 W/(m^2*K)
ρ_m	Density of water	1000 kg/m^3
C_m	Heat capacity of water	4182 J/(kg*K)
k_m	Thermal conductivity of water	0.6 W/(m*K)
S	Surface area of GNR	depends on the size of the GNR
V	Volume of GNR	depends on the size of the GNR
power	maximum power during a period	The conversion relationship between maximum power and energy in one period is as follows:<br>Maximum power= $\frac{\text{energy in one period}}{\sqrt{2\pi}t_\sigma}$<br>energy in one period is 28/3pJ for most of case in paper

4. References

[1] Gole, A.; Murphy, C.J. Seed-mediated synthesis of gold nanorods: role of the size and nature of the seed. *Chem. Mater.* **2004,** 16, 3633-3640.

[2] Kumar, P.; Nagarajan, A.; Uchil, P.D. Analysis of cell viability by the MTT assay. *Cold spring harbor protocols,* **2018,** pdb-prot095505.

[3] Johnson, P.B.; Christy, R.W. Optical Constants of the Noble Metals. *Phys. Rev. B* **1972,** 6, 4370–4379.
